# Evaluation of uptake and attitude to voluntary counseling and testing among health care professional students in Kilimanjaro region, Tanzania

**DOI:** 10.1186/1471-2458-9-128

**Published:** 2009-05-09

**Authors:** Mgosha P Charles, Eliningaya J Kweka, Aneth M Mahande, Longin R Barongo, Seif Shekalaghe, Hassan M Nkya, Asanterabi Lowassa, Michael J Mahande

**Affiliations:** 1 Ministry of Health and Social Welfare, National AIDS Control Programme division, PO Box 11857, Dar es Salaam, Tanzania; 2 Tropical Pesticides Research Institute, Division of Livestock and Human Disease vector control, PO Box 3024, Arusha, Tanzania; 3 KCM College of Tumaini University, Community Health Department, PO Box 2240, Moshi, Tanzania; 4 National University of Rwanda, Research Department, BP 117, Butare, Rwanda; 5 Tanzania Wildlife Research Institute, Research Department, PO Box 661, Arusha Tanzania

## Abstract

**Background:**

Voluntary counseling and testing (VCT) is a corner stone for successful implementation of prevention, care and support services among HIV negative and positive individuals. VCT is also perceived to be an effective strategy in risk reduction among sexually active young people.. This study aimed to assess the acceptability of VCT and its actual uptake among young health care professional students at KCM College of Tumaini University and Allied health schools.

**Methods:**

This was a cross-sectional study. A structured questionnaire was used among health care professional students aged 18–25 years who were enrolled in degrees, diplomas and certificates courses at Kilimanjaro Christian Medical College and all other Allied health schools

**Results:**

A total of 309 students were recruited, among these 197 (63.8%) were females. All respondents were aware of the benefits of VCT. Only 107 (34.6%) of students have had VCT done previously. About 59 (19.1%) of the students had negative for health care professional to attend VCT. Risk perception among the students was low (37.2%) even though they were found to have higher risk behaviors that predispose them to get HIV infection.

**Conclusion:**

Awareness of VCT services and willingness to test is high among students; however its uptake is low. In order to promote these services, a comprehensive training module on VCT needs to be included in their training curricula. In particular, more emphasis should focus on the benefits of VCT and to help the students to internalize the risk of HIV so that they can take preventive measures.

## Background

Human Immunodeficiency Virus/Acquired Immunodeficiency Syndrome (HIV/AIDS) is a killer disease affecting all age groups from infants to old people [1]. HIV/AIDS has killed more than 3 million adults and children in the year 2005 and it is estimated that 5 million adults and children acquired the infection to make the number of adult and children with HIV/AIDS to be 42 million worldwide [2].

Sub-Saharan Africa remains the most affected region in the world with an estimate of 22.5 million people living with HIV. Approximately 1.7 million new infections occurred in sub-Saharan Africa in the year 2007 [1]. Ten million young people aged 15–24 years and almost 3 million children under 15 years are living with HIV [1].

Sub-Saharan Africa is the region with the highest overall HIV sero-prevalence, especially among adults (15–49 years) population. However this varies between countries, ranging from less than 2% to above 15%. For example, in Somalia and Gambia the prevalence is below 2%. In other countries HIV prevalence varies; Zambia (20%), Botswana (38.8%), Lesotho (31.5%), Swaziland (33.4%), Central African Republic (12.4%), Nigeria (5.8%), Kenya (15%) and Uganda is (5%) [1].

Tanzania was estimated to have about 2.2 million adults living with HIV/AIDS, among which 15% are in 15–24 year age groups and 60% of all new infection occurs in this age group. The overall prevalence of HIV infection among blood donors in 2004 was 7.7% while that in the Ante-natal clinic was 8.4% [3].

Currently, there is no cure or vaccine for HIV/AIDS; however, the provision of Antiretro viral drugs and positive prevention strategies helps to prolong life for those who are already infected. Many countries have been trying to take many different approaches in an attempt to slow the spread of HIV infection and minimize its impact on the individual, family and society. Among these strategies include; voluntary counseling and testing (VCT), provider initiated counseling and testing (PICT), diagnosis of HIV in infants and young children, family care and partner testing and counseling based on index care, condom promotion and provision, detection and management of sexually transmitted infections, safer sex and risk reduction counseling, male circumcision, targeted interventions for sex workers and men who have sex with men (homosexual practice). Others include; occupational and non occupational post exposure prophylaxis, family planning and counseling, antiretroviral medicines for preventing HIV infection in infants, treatment, care, support for pregnant HIV positive women, infant feeding counseling and support, prevention of HIV from mothers to children and prevention of HIV (and tuberculosis) transmission in health care settings (Infection control), blood safety, safe injections and use of standard precautions. All these strategies emphasizes on behavior change and risk reduction behavior which both adult and youth have shown to have positive response to VCT [4]

In Tanzania voluntary counseling and testing activities started in 1997, by the end of April 2008 about 5,319,247 clients were counseled and tested for HIV in 1643 HIV voluntary counseling and testing centers in the country [5].

Health care professional students are not spared with the increased HIV infections because majority of them fall under the age group which are at risk of HIV infection and occupational exposure [6]. In addition, the public expect the medical professionals to be seen as a role model in health care seeking behaviour including VCT.

It was the objective of this study to assess the acceptability of VCT among the vulnerable group of health care professional students (18–25 years) at KCM College of Tumaini University and other health allied schools in the campus in Moshi town. The information that generated from this study for will be useful to health policy makers in developing HIV/AIDS interventions, curriculum development and provision of youth friendly services

### Methodology

#### Study Design

A cross-sectional survey was conducted between April and May 2005.

#### Study Area

The study was conducted at KCM College and other affiliated allied Health Schools at Kilimanjaro Christian Medical Centre. The Kilimanjaro Christian Medical College is incorporated in the Kilimanjaro Christian Medical Center, a research and teaching hospital. It is one among the four-referral hospitals in the country being situated in Moshi urban district, which is one of the six districts of Kilimanjaro region in North Eastern part of Tanzania.

There are about eight voluntary counseling and testing centers in Moshi Municipality, most of them located in town, and these includes, Kinshai, Langani, KCMC-Mbuyuni, Mawenzi Hospital, Rainbow, Pasua Health Center, KCMC and "KIWAKKUKI". KIWAKKUKI is a female non governmental organization which stands for "women association to fight against HIV/AIDS Kilimanjaro

### Study Population

This study targeted health care professional students undertaking degrees, diplomas and certificates courses at KCM College and all other Allied Health Schools at Kilimanjaro Christian Medical Center campus, student aged 18–25 years. (According to Tanzanian law, an adult is said to be anybody aged 18 years and above. These can give consent on their own without soliciting permission from parents or teachers).

### Sampling Frame

We only aimed to recruit students within the ages 18 to 25 years from 18 Schools in KCMC campus including KCM College. Therefore, all students aged between 18–25 years were eligible for this study, those who consented for the study, those studying at KCM College and other Allied Health Schools at KCMC campus and who were able to speak either Kiswahili or English language. The exclusion criteria for the study were; students who were either below or above 18–25 years age group, student's who did not consent to participate in the study, students who were not from KCMC or from any of the Allied Health Schools at KCMC campus and those Students who could not communicate either in English or in Kiswahili language

Out of 780 total number of KCMC students, only 420 students were eligible for this study. A convenience sampling method was used to select the study participants. Only students who were available during the class sessions were selected. The author administered questionnaires to all eligible participants. The questionnaires were retrieved from 309 students, the rest 11(2.6%) did not return the questionnaire to the author.

### Data Collection

Structured questionnaire was prepared to capture the relevant information based on the study objective. Questionnaires are initially administered to similar respondents in a non-participating institution for validation.

The investigator visited each school in the study area. A specific day and time for each school was arranged in order to meet students in their respective classrooms in order explain the study objective.

#### Data Management and Analysis

Data base was made in MS-access. Data entry was done in duplicate for validation (double entry). Before analysis, data were cross-checked for entry error and range checks. Analysis was done using SPSS version 15.0 for windows. Descriptive statistics were obtained for different quantitative variables. Frequencies and percentages were used to present categorical variables. Furthermore, cross tabulations were done to determine some significance of associations between variables, where Chi Squared test and their respective p-values were calculated. The p = 0.05 (2-tailed) was used as a cut-off point to test statistical significances between variables compared.

### Ethical Issues

Ethical clearance was obtained from KCMC ethical committee.

Permission to conduct this study was obtained from the Provost of KCM College of the Tumaini University and Principals of all Allied Health sciences schools at KCMC campus. Individual consents were sought from the study participants before starting the study; participants were requested to sign the consent forms after they had understood the study aims and before answering the questions. Participants were also informed that participation was on voluntary basis. Confidentiality was assured, where anonymous questionnaires were used. Students who were not selected in the study were told the reason for their ineligibility.

## Results

### Demographic characteristic of the study population

A total of 309 (96.6%) health care professional students from different health schools at KCMC were enrolled in the study. The mean age and standard deviation for male and female were 23.7 ± 1.46 and 23.3 ± 1.68 years respectively. There were 112 (36.2%) males and 197 (63.8%) females, giving a sex ratio of 1:1.8. Most of the study participants were single 294 (95%).

Majority of the respondents 154 (49.8%) were within the 24–25 years of age. The distribution of students in different age groups is shown in Table 1. Most of the students, 123 (39.8%) were Catholics followed by Protestants 105 (34.0%), Muslim, 44 (14.2%) and few 37 (12.0%) belonged to other denominations. The participants education levels ranged from; Certificate 35 (11.3%), ordinary diploma 174 (56.3%) to first-degree 100 (32.4%).

**Table 1:** Age group at first sexual exposure by gender

Age group (years)	no (%)			
	Male	Female	χ^2^	p Value
<10 years	13(11.7)	12 (7.0)	13.8	0.02
				
10–12 years	7 (6.3)	18 (9.2)		
13–14 years	6 (5.4)	20(10.2)		
15–17 years	14(12.6)	44(22.3)		
≥ 18 years	75(62.5)	96(48.7)		
Never had sex	2 (1.8)	7(3.6)		
Total	112(100)	197(100)		

### Risk assessment

A higher proportion of males 110 (98.2%) reported to have had sexual experience compared to 190 (96.4%) females. Among the 309 respondents in the sexually active group, 114 (36.9%) reported to have contracted sexually transmitted infections (STIs) previously. A lower proportion of male students 46 (40.4%) ever contracted STIs compared to their female counterpart 68 (59.6%). However, this difference was not statistically significant (χ^2 ^= 1.32, p = 0.25).

Among 169 (54.6%) of the respondents who provided information about alcohol consumption, 146 (47.2%) reported to have had sex under the influence of alcohol. A higher proportion of females 98 (67.1%) reported sexual exposed under the influence of alcohol as compared to males 48 (32.9%).

Females were two times more likely to be at high risk compared to their males counterpart (χ^2 ^= 6.7, p = 0.01). In contrast, females were more likely to have one current sexual partner compared to males. This difference was statistically significant (χ^2 ^= 6.17, p = 0.01).

The reported age of sexual debut ranged between 6 to 20 years with the mean age of 11.26 ± 3.6 years (Table 1). There was no difference in mean age at sexual debut between the two sexes.

About 25% of the sexually active group had experienced sexual intercourse by the age of 9 years, 50% by the age of 12 years and 75% by the age of 14 years. Males reported to engage in sex more than females. However, the difference was not statistically significant (χ^2 ^= 2.8, p = 0.1). Males were more likely to have sex after 18 years of age compared to females. The difference was statistically significant with (χ^2 ^= 4.8, p = 0.03).

Many students, 250 (83.3%) reported to have their first sexual intercourse with their boyfriend or girl friends, 21 (7.0%) had casual sex, 15 (5.0%) had sex with their relatives, and 14 (4.7%) revealed that they were raped. Those who were raped, 11 (78.6%) were females and 3(21.4%) males.

Students in the age group 24–25 years had slightly higher proportion (49.8%) of being sexually exposed compared to other age groups. In addition, the age group 22–23 years (35.0%) had sexual exposure while for the youngest age group 18–21 years only (15.2%) had sexual exposure in their lifetime. However the differences were not statistically significant (χ^2 ^= 2.5, p = 0.28).

The characteristics of sexually active participants are shown in table 2. Among 300 students who responded to the question on the number of their sexual partners, majority 196(65.3%) reported having multiple sexual partners; female were 1.9 times more likely to have multiple sexual partners compared to males (χ^2 ^= 6.17, P = 0.01).

**Table 2:** Distribution of the sexually active respondents by gender and the number of partners they had in their lifetime.

Number of sexual partners	No (%)			
	Males	Females	χ^2 ^=	p Value
1	48(42.9)	56(28.4)	6.9	0.03
>1	62(55.4)	134(68.0)		
				
Never had partner	2(1.8)	7(3.6)		
**Total**	**112 (100)**	**197 (100)**		

Regarding the risk perception, students were asked to grade their risk as being high, low and no risk (Table 3). Majority of them; 194 (62.8%) thought to be at low risk of contracting HIV and the rest 115 (37.2%) considered themselves to be at high risk. However, students who perceived to have low risk of contrasting, 190 (96.9%) had sex already and only 6 (3.1%) have never had sex. The risk perception was not statistically significantly associated with sexual exposure (χ^2 ^– Fishers exact = 0.06, p = 0.81).

**Table 3:** Association between sexual exposure and perceived risk

**Perceived risk**	**Ever sexual Exposure**	**History of STI**	**History of Narcotic drugs**	**History of alcohol influencing sex**
	Yes N (%)	Yes N (%)	Yes N (%)	Yes N (%)

High	112 (36.7)	54(47.0)	65(78.3)	75(48.1)

Low	118(63.3)	60 (53.0)	18 (21.7)	81 (51.9)

Total	300 (100)	114 (100)	83 (100)	156 (100)

Statistical tests	χ^2^(Fishers exact test) = 0.06; d = 1, p = 1	χ^2 ^= 7.29, d = 1, p = 0.007	χ^2 ^= 79.64, d = 1, p ≤ 0.0001	χ^2 ^= 14.98, d = 1, p = 0.0001

Regarding STIs, 60 (53.0%) of the students perceived to have low risk of contracting HIV despite the history of STIs as compared to 134 (69.1%) students who never had history of STIs. About 18 (21.7%) of the students perceived to have low risk of contracting HIV despite the history of having sexual intercourse after they had taken Narcotic drugs compared to 176 (77.9%) students who never took Narcotic drugs but had sexual intercourse either. The risk perception and history of STI was statistically significantly related (χ^2 ^= 7.29, p = 0.007). Similarly, use of narcotic drugs was significantly associated with risk perception (χ^2 ^= 79.64, p =< 0.001)

More than half, 81 (51.9%) of the students perceived to have low risk of HIV despite the history of having sexual intercourse under the influence of alcohol compared to 113 (73.9%) who never sex under the influence of alcohol. There was a statistical significant difference in risk perception of acquiring HIV for those who perceived high risk due to sexual exposure 110 (36.7%) as compared to 3 (33.3%) who never had sex. (χ^2 ^= 14.98, P ≤ 0.001).

Those who have not perceived higher risk due to history of STIs 54(47.0%) reported more of contracting HIV as compared to 60 (30.9%) students who never had history of STIs (χ^2 ^= 7.29, P = 0.007). There was a statistically significant difference in risks of contracting HIV for 65 (78.3%) students who perceived higher risk following the use of Narcotic drugs compared to 50 (22.1%) students who never abuse Narcotics. (χ^2 ^= 79.64, P ≤ 0.001).

We also observed the statistical significant difference in risk perception. Students who reported to have sex under influence of alcohol 75 (48.1%) were perceived to have high risk compared to those who never had sex under the influence of alcohol 40 (26.1%). The difference was statistically significant (χ^2 ^= 14.98, P < 0.0001).

### Awareness of voluntary counseling and testing services

All students 309 have heard about HIV voluntary counseling and testing service. There was no statistically significant difference between males and females regarding correct information and Knowledge on benefits of VCT (χ^2 ^= 0.8, p = 0.4).

**Figure 1:**
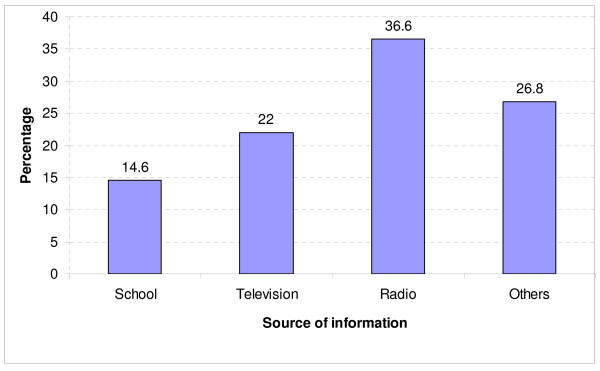
**Source of information about VCT services**.

Students had multiple responses with regard to source of information about VCT. More than half (58.6%) heard it from mass media (Radio and Television) and others have heard it from other sources like taught at schools, friends, church seminars, and through visiting VCT centers as shown in Figure 1.

About 100 (32.4%) Medical degree, 93 (30.1%) Nursing, 35 (11.3%) physiotherapist and 81 (26.2%) others like Medical record technicians, pharmaceutical assistants, occupational therapist, Optmetricians and TATCOT students were aware of VCT programmes. There was no statistically significant difference between VCT awareness by gender, age groups, program enrolled and religion of the students.

When asked to whom is VCT targeted, 240 (77.7%) reported is for anybody, 42 (13.6%) is for adults only, and others 27 (8.7%) thought that it's for infected people. Majority of students 230 (74.4%) knew where to go for VCT services in Moshi.

On the question about the benefits of VCT, the most common responses were, knew HIV sero-status, 138(44.7%) and acquiring HIV knowledge 64 (20.7%). Other benefits of VCT which were cited are enhancement of risk reduction 21(6.8%); treatment and support 15 (4.9%), being able to plan for future 35 (11.3%) and only 36 (11.7%) don't know the benefits of VCT.

### Factors associated with having had VCT

Factors associated with VCT uptake has shown in Table 4. Of 309 students who heard about VCT, only 107 (34.6%) reported to have attended voluntary counseling and testing centers.

**Table 4:** Factors associated with VCT attendance

**Factors**	Have you ever attended VCT (N (%))			
	Yes	No	χ^2^	p-value
**Religion**				
Protestant	36(29.3)	87(70.7)	5.6	0.13
Catholic	39(37.1)	66(62.9)		
Muslims	21(47.7)	23(52.3)		
Others	11(29.7)	26(70.3)		
				
**Professional groups**				
Medical students (MD)	35(32.7)	65(32.2)	0.98	0.80
Nursing	33(30.8)	60(29.7)		
Physiotherapist	14(13.1)	21(10.4)		
Others	25(23.4)	56(27.7)		
				
**Age group**				
18–21	16(15)	31(15.3)	3.9	0.14
22–23	30(28)	78(38.6)		
24–25	61(57)	93(46)		
				
**Gender**				
Male	38(35.5)	74(36.6)	0.04	0.85
Female	69(64.5)	128(63.4)		
				
**Ever had sex**				
Yes	106(99.1)	194(96)	2.26	0.17
No	1(0.9)	8(4.0)		

Of 123 number of Catholics, 36 (29.3%) reported having had VCT, 39/105 (37.1%) of Protestants, 21/44 (47.7%) of Muslims and 11/37 (29.2% others Religion was not significantly associated with VCT uptake (χ^2 ^= 5.6, p = 0.13).

There was no statistically significant difference between VCT attendance with regards to gender, age groups, sexual exposure and cadre of the students (Table 4).

About 202(65.4%) students have never attended VCT. More than half 107(53.0%) mentioned that they don't see the need, 70(34.6%) mentioned fear being the obstacle, 20(9.9%) mentioned that they trust themselves and 5 (2.5%) had other reasons.

### Attitude toward VCT

Some questions were asked in order to understand the attitude of students towards VCT services These includes; importance of VCT, reasons for attending VCT, willingness to test and if ARV be should freely available.

The majority 290(93.9%) had the opinion that VCT is important to enable a person knowing his/her sero-status. However, very few (7.1%) had a misconception that VCT pre-dispose them to early sexual matters. About 59(19.1%) had negative attitude for health care professional students to attend VCT. More than half 197(63.8%) had positive attitude towards attending VCT and 53(17.1%) were undecided. The majority, 264(85.4%) of the students were willing test for HIV if requested.

Almost all students 294(95.1%) agreed that VCT services should be provided free of charge to students. The majority 265(85.8%) agreed that students who are infected with HIV should be freely provided with antiretroviral drugs and only 44(14.2%) were not sure.

Regarding the preferred model of provision of VCT services to students. The majority 275(89.0%) preferred college based VCT, although a large proportion 224(72.5%) reported that the service should be integrated into youth programs such as STIs and family planning. In addition, participants recommended that VCT services should better be given during youth activities like sports, drama and festival. However, the majority 202(65.4%) reported that VCT services for young people should not be mixed with adults and more than a quarter, 107(34.6%) suggested that VCT services should be carried out at private centers in town.

## Discussion

In this study we assessed factors related to attitude and uptake of VCT among health care professional students at KCMC in Kilimanjaro region.

### Risk assessment

We observed majority of students had sexual debut at an earlier age from six years with mean age of 11 years. These findings are in line with findings from other studies in Africa [7-9] and that from America which reported sexual debut to be at 16 and 17 years for males and females respectively [10].

The sexual experience was found to differ between males and females and with different age of sexual debut. Male students were found to be exposed earlier to sex compared to their female counterpart. Similarly older age groups have shown to be more exposed than the youngest group. These differences could be explained by the fact that males were more likely to be explicit about their sexual experiences and that because of early sexual debut older students were found to have more experience than young one as reported else where [7] in Tanga. Female students were found to have multiple sexual partners compared to male students, this could be probably because females are looking for gifts and money so it's possible that females get what they need; they might be tempted to have a number of partners or change of partners. For males, they have to restrict themselves from having many partners, because they have to give something to females. These results are similar with the study report done in Tanga; young females were likely to have multiple sexual partners due to economic reasons [7].

Youths are at risk contracting HIV infections. HIV risk perception is an important determinant of behaviors change and therefore complementing HIV/STI's preventive measures. In this study majority of students do not perceive themselves as being at risk of contracting HIV. The low perceived risk of HIV among youths was also found in other studies [7,11-13]. Compared to other studies where the significant proportion 24% of young people perceived themselves to have had low or no risk [7]. The highest percentage found in this study (62%) compared to that in Tanga (24%) could be due to different study population, Tanga study used youth not at school while in this study we used youth in Health schools. Low perceived risk among youths is reported elsewhere in Africa Kenya, Uganda, South Africa, and Zimbabwe and in USA [13].

### Awareness, knowledge and benefit of VCT services

Although in this study the highest proportion among students from different courses was from medical, in general majority of students were aware and knowledgeable about VCT. This finding is in agreement with other studies in Kenya, Uganda and Tanzania [11,14,15] respectively. Mass media played a great role as a source of information on the awareness of VCT in general. Most of students reported to have obtained information from radio and television which are readily available at their homes and within their hostels at KCMC. Majority of students failed to understand several important benefits of VCT services. Benefits such as change of behavior, getting support and early treatment for infected and future planning and risk reduction were mentioned by only few students which is the case to other previously published studies [16,17]. This indicates how the relationship between knowledge and awareness and other behavioural factors is complex, since there is no direct relationship.

### VCT uptake and factors associated with

The low uptake of VCT services to majority of the students was a remarkable observation in this study. This study observed slight higher VCT service uptake (34.6%) among students compared to other studies in Uganda (10%) [14] and in Zambia (14.6%) [17]. This might be explained by the poor risk perception and lack of knowledge regarding the VCT benefits.

Religion is important factor reported in several studies regarding the use of condom [11]. Similarly, in this study religion has great influence on the VCT uptake services. Even though the Catholics were the majority for the attendance of VCT, but they have shown the least proportion of attendance to voluntary counseling and testing services as compared to other denominations. This could be explained by the negative attitudes towards condom use and other contraceptive methods offered during VCT services attributed by the religious believe.

Low perception of risk to HIV among youth has been cited in several studies [11,14]. This has also been mentioned as a factor for low VCT uptake in this study.

### Attitude and Uptake of VCT services among students

Perceived risk of HIV and benefit of VCT services have a major influence on the attitude and uptake of VCT among youths and other people [18]. In this study majority of students (85.4%) expressed their willingness to test for HIV which correlates with other studies done among youth in Kenya and Uganda [15] and is explained that willingness and practice may not always support each other since many people seems to be willing but those who actually go for the services are few [15]. Reasons to this may be those already mentioned earlier for example lack of youths perceiving their healthy risk in general and lack of perceived benefits of VCT [16,17] but also the important place where the VCT services is being given and who actually gives the service.

Young people are not particularly interested in health issues; they invest their time and interest more in areas like school, job training, sports and media [11]. It was suggested that special efforts have to be done to attract students and other youth in VCT programmes [16]. In this study youths preferred different models of VCT services for young people for example VCT should to be separated from the adults and also VCT services should be carried out in private centers in town by experienced and preferably adults people.

Our findings suggests that health education and health information about HIV and VCT services need to be provided and encouraged to health care professional students, with more emphases on the important and benefits of this services. However, this call for establishment for VCT centers in their respective settings. Findings from this study also urges program managers and practitioners to consider VCT as an important component for care and treatment for HIV/AIDS, VCT services should be provided free of charge and the effort of obtaining drugs should be made so that those who test positive need to access treatment, care and support.

## Conclusion

Awareness of VCT services is high among health care profession students at KCM College. However, knowledge on most important benefits of VCT services is still lacking.

Our study has revealed the low perceived risk of HIV among the studied population and this might be one of the contributing factors to their low attendance for VCT services. However, most of the students showed positive attitude towards VCT and reported willing to test if they were given opportunity. Our findings suggest that students prefer a different mode of VCT apart from the existing one.

Youth friendly VCT services need to be established to attract this vulnerable group because they are at risk and mostly affected with HIV/AIDS but the scaring thing is that students themselves do not know about that.

This study have the following limitations: Findings from this study can not be generalized to the whole population of the young people because the study involved only those young people who are in school/colleges and not those who have not attended schools/colleges.

There is a possibility of having under reporting of certain information because the study was done within KCMC premises so students' might fear to disclose some information. Some information needed a recall memory so there could be a recall bias. More research is needed to see on how VCT services could be promoted within this special group of youths.

## Competing interests

The authors declare that they have no competing interests.

## Authors' contributions

MPC, EJK, LB and MJM designed the study and performed statistical data analysis. AMM and SS are expert in community based research and AL and HMN are a behavioral scientists; both contributed to the design of the study, development of the data collection instruments and standard operating procedures, data analysis, and manuscript preparation. All authors read and accepted the final version for submission.

## Pre-publication history

The pre-publication history for this paper can be accessed here:


